# Orchard Sports Injury and Illness Classification System (OSIICS) version 16: Updated female athlete codes and Italian and Spanish translations

**DOI:** 10.1016/j.jsampl.2025.100119

**Published:** 2025-10-31

**Authors:** John W. Orchard, Isabel S. Moore, Federico Genovesi, Molly McCarthy-Ryan, Daniel Martinez, Ebonie Rio, Kay Crossley, Jessica J. Orchard, Ben Clarsen, Margo Mountjoy

**Affiliations:** a School of Public Health, University of Sydney, Sydney, Australia; b Cardiff School of Sport and Health Sciences, Cardiff Metropolitan University, Cardiff, UK; c Manchester City Football Club, Manchester, UK; d Argentine Football Association (AFA), Buenos Aries, Argentina; e La Trobe Sport and Exercise Medicine Research Centre, La Trobe University, Melbourne, VIC 3083, Australia; f Oslo Sports Trauma Research Centre, Oslo, Norway; g Department of Family Medicine, McMaster University, Hamilton L8S 4L8, Canada

**Keywords:** Breast, Women, Spanish, Italian, Classification, Coding

## Abstract

**Background:**

The Orchard Sports Injury and Illness Classification System (OSIICS) requires regular update to; stay relevant to changes required for optimal code recording; to add additional language translations as they become available and add female-specific codes to reflect contemporary clinical diagnosis and the increase in women's sport participation.

**Methods:**

Codes were added to the existing version 15 after the assembly of an ad hoc panel of co-authors. Being a revision that only added codes without changing any structure, informal methodology, primarily conducted by email discussion amongst the author group, was used to resolve issues. Two authors who were native Italian and Spanish speakers, and fluent in English were used to create the respective language translations.

**Results:**

The area of greatest deficiency for OSIICS versions 13–15 was in coding for breast conditions. To fit in with the existing consensus categories, the injury format used was CxBxx with a third character B to signify breast within the chest region. For breast illness/medical disorders, the coding format was MxxBx

**Conclusion:**

Although consideration was given to creating a new, standalone category for breast injuries, due to the formalised alignment between OSIICS and Sports Medicine Diagnostic Coding System it was decided such a change requires the IOC consensus panel to re-convene. Additionally, the medical/illness categories need consideration of whether breast disorders best fit in the endocrine or genitourinary system, or if an expanded medical category is needed for female reproductive medical conditions.

## 1 Introduction

The Orchard Sports Injury and Illness Classification System (OSIICS) is one of two sports injury classification systems recognised by the International Olympic Committee (IOC). The other system is the Sports Medicine Diagnostic Coding System (SMDCS) [1]. The most recent IOC injury and illness surveillance consensus conference update in 2019-2020 prompted an update of the OSIICS (version 13) [2]. Subsequently, another update was published in 2024 (version 15) to include mental health conditions, sports cardiology, concussion sub-types, infectious diseases and skin and eye conditions [3]. Regular version updates of the OSIICS are encouraged to include additional coding and translations into non-English languages [4].

The first version of OSICS (precursor to OSIICS) was published in 1993 [5], with a focus on sports injuries (rather than illnesses) and male athletes, given that it was originally written to code injuries for surveillance in the (male) Australian Football League. Version 10 (2007) was the first attempt to include a substantial number of illness codes [6]. In 2020 the name OSICS was changed to OSIICS to formally recognise the increasing predominance of illness. The 2020 IOC consensus update had a primary aim of resolving challenging coding decisions between OSIICS and the SMDCS with a consensus methodology [1,2]. This was achieved by agreeing on body region and injury type categorisations that would be consistent between systems. An example of this was whether Achilles tendon injuries should be categorised as “Ankle region” or “Calf region” injuries. There was a regional argument in favour of ankle (that is, the Achilles tendon is close to the ankle) but a system argument in favour of the calf (that is, the Achilles tendon is the tendon associated with the calf muscle group, suggesting that calf and Achilles injuries should belong in the same body region). A consensus was needed between classification systems to ensure research reporting body region injury rates using different classification systems can still be accurately compared. In the case of Achilles tendon injuries, the consensus decision was resolved in favour of considering them to belong in the calf region rather than the ankle region.

Elite level women's sport was relatively neglected in terms of sports injury surveillance until recently. With a greater number of sports and sports clubs funding the professionalisation of women's sport (e.g., women's soccer, rugby union, ice hockey), sports injury and illness surveillance is becoming more frequently conducted with female athletes [7,8]. Recent versions of OSIICS (14 and 15) have looked to include female-specific diagnoses to accommodate the growth in female injury and illness surveillance. With the newly described female athlete health domains [9], parity in women's participation at the Olympic Games (2024), and increasing mother-athlete participation in elite level sport, there is a need to incorporate more female-specific diagnoses particularly for domains that have relied on broad, undiagnosed injury and illness prevalence rates such as perinatal health (pregnancy and postpartum) [10], breast health [[11], [12], [13]], menstrual health and pelvic floor health [14,15].

There is a trade-off between greater diagnostic specificity and simplicity of the coding systems. Updates need to balance the benefits of more codes, which provide greater opportunity to code a specific diagnosis against being able to efficiently navigate code options. Currently, the OSIICS has over 1500 coding options, which is more detailed than SMDCS but far less detailed with respect to illness than the International Classification of Diseases (ICD)-11 [16] (over 30,000 codes). However, a strength of the OSIICS and SMDCS is the number of sports injury codes, which the ICD lacks as it focuses on illness specifically. Improving diagnostic specificity of the OSIICS to accommodate female athlete domain considerations will enable better identification of female-specific health problems and support the development of athlete welfare strategies. Therefore, this paper presents OSIICS Version 16, an update to include additional female-specific diagnoses and Italian and Spanish translations.

## 2 Methods

This current update (version 16) aimed to achieve:

(1) Inclusion of additional female athlete specific codes, supported by emerging literature in this field;
(2) Ad hoc inclusion of further diagnoses that were requested since the release of version 15; and
(3) Translation into Italian and Spanish (the former updating the version 14 translation).

The co-authors were chosen from the author groups of versions 13–15 (JWO, MM, KC, ER, JJO) along with co-authors who led the additional female athlete specific codes (ISM, MMR) and translations into Italian and Spanish (FG and DM).

Prior to reconvening the IOC injury surveillance full panel of experts needed for major updates to the OSIICS, minor updates were decided by broad consensus of the author group. The methods included additional female athlete specific codes being generated by reviewing recent literature and discussions with pelvic health specialists and sports medicine musculoskeletal physiotherapists. Those codes were sent to all authors and informal debating via email correspondence about the structure of the new codes, with care taken to avoid duplicates of existing codes was undertaken. Where disputes arose, we deferred to the IOC consensus statement [1] initially, with casting vote falling to the primary author (JWO). There were no formal processes (such as Delphi) used to create the new codes. The language translations were undertaken by FG (Italian) and DM (Spanish) who are both fluent in their native language and English.

## 3 Results

Table 1 details additional female athlete diagnoses that were felt to not previously be adequately covered in version 15. Broadly, the diagnoses added cover the following female athlete domains [9]: perinatal health (including childbirth), breast health, pelvic floor health and mental health. To accommodate prospective injury and illness surveillance of athletes going through pregnancy, childbirth and postpartum, codes relating to mode of delivery, common perinatal mental health problems, breastfeeding-related health problems and pregnancy loss were added.

**Table 1:** Female athlete additional codes added to OSIICS 16.

OSIICS16 code	Diagnosis
CIBC	Breast or torso chaffing (friction injury)
CIBX	Breast abrasion
CKBX	Breast laceration
CPB1	Exercise-related breast pain
CVB1	Breast haematoma/trauma
CVB2	Severe breast haematoma associated with rapid expansion in breast size
CVB3	Breast fat necrosis or oil cyst delayed presentation related to breast trauma
CVB4	Structural breast deformity delayed presentation related to breast trauma
CVBM	Milk-duct injury
CVBR	Breast implant rupture
MSAA	Antenatal anxiety
MSAD	Antenatal depression
MUBB	Ovarian cyst (benign)
MUBC	Cervical/uterine cancer
MUBO	Ovarian cancer
MUZAB	Abortion
MUZC	Postpartum ceasarean section
MUZL	Vulvodynia
MUZM	Miscarriage
MUZP1	Perineal tear (grade 1–2)
MUZP3	Perineal tear (grade 3–4)
MUZPE	Ectopic pregnancy
MUZQ	Pelvic organ prolapse
MUZV	Postpartum vaginal delivery
MYSBT	Mastalgia (cyclical breast tenderness)

The expert content authors on women's health requested creating a new body region “Breast” (with coding structure BXxx, B indicating body area Breast, X being the tissue type and xx being the further detail). This request was rejected for version 16, primarily on the basis that it would have created a different body category to the consensus agreements. However, it was felt that it was important to be able to categorise breast injuries easily. Consequently, breast-related injury codes were included with the following format instead - CXBxx - meaning they are included in Chest region.

These codes have been added to accommodate the recent breast tissue injury categorisation of contact-related sport injuries [17], adapted from seatbelt trauma classification [18], as well as other breast injuries. In particularly, the OSIICS have been updated accounting for the four breast tissue injury categories (1A-B, 2A-B). These categories supersede the previous single breast haematoma categorisation, whose code “CVBH” has been revised to CXBXX (breast tissue injury category 1A). Other breast health problems, such as breast implant rupture, exercise-related pain, mastalgia [19] (cyclical breast tenderness), abrasion, laceration and chaffing (frictional) injuries due to equipment (e.g., sports bras) have also been added.

Pelvic health within sports medicine is a growing field [20]. There were several pelvic floor dysfunction codes from previous versions, but these have been expanded and updated to include birth-related trauma.

Further ad hoc codes that were suggested and agreed by co-authors as lacking in previous versions have been included (Table 2). A supplementary group of Tables included as part of an online spreadsheet details the full English set of codes for version 16, along with the Spanish and Italian translations.

**Table 2:** Further additional codes added to OSIICS 16.

OSIICS16 code	Diagnosis
FRE	Ruptured toe extensor tendon
FSON	Navicular bone oedema
KL6	Knee medial patellofemoral ligament injury
MESC	Ear wax/cerumen
MNSV	Vertigo, unknown cause
MRYL	Lupus/systemic lupus erythematosus (SLE)
MUBT	Testicular cancer
MUJE	Epididymal cyst
MYNG	Gynaecomastia
NQF	Cervical Spine facet joint synovitis/pain
OP1	Exertional abdominal pain
OPS	Runner's stitch
PLF	Finger joint sprain (PIP and DIP joints)
PM1	Hand intrinsic muscle injury
PTP2	Flexor A2 pulley injury
PTP4	Flexor A4 pulley injury
QRP	Plantaris tendon rupture
QSOF	Fibula bone oedema
QSOT	Tibial bone oedema
QTAP	Achilles paratenon injury
QTPT	Plantaris tendinopathy
RSOR	Bone oedema radius
RSOU	Bone oedema ulna
RSXR	Radial stress fracture involving cortex
RSXU	Ulna stress fracture involving cortex
TVML	Morel-Lavallée lesion, hip/thigh

## 4 Discussion

This update to OSIICS includes more female specific codes, opens a debate about possible future category changes and, finally, includes updated Italian and Spanish translations.

Whilst there were arguments for and against structural modifications for body region given recent calls to enhance surveillance of breast-related health problems [21], the majority agreed that it was inappropriate to introduce changes that would alter the consensus categorisations of the 2020 version. Instead, we suggest consideration of this topic at the next full sitting of the IOC injury and illness consensus panel.

The primary issue that needed resolution in this update is whether an additional body region should be created to signify breast injuries (and illnesses) as a separate body region (system). Although we resolved not to create a unique body region for breast injuries, the arguments in favour and against are presented for consideration at future sports injury coding consensus conferences.

The major argument for a distinct category for the breast is that it is part of female reproductive system but anatomically located remotely from the other female reproductive parts, which are found in the abdomen/pelvis/groin regions. Not only are the IOC consensus injury categories regional, but the medical categories are also challenging for breast disorders, perhaps slanting more towards the “endocrine” system rather than “genitourinary” system (which are the two closest options in the IOC medical categories). If breast injuries are included in the chest region (current regional classification) then these female-specific diagnoses are somewhat hidden alongside dissimilar injuries (e.g. sternum and rib bruising and fractures). This is problematic for several reasons. Firstly, the breasts have health considerations that cross several female athlete health domains [22] (e.g., mastalgia would be categorised as menstrual health, mastitis would be categorised as postpartum) and require clear identification of female-specific diagnoses [9]. Secondly, being able to make evidence-informed decisions regarding equipment, which can affect the Laws governing sports (e.g., breast protection regulations), requires breast injuries to be differentiated from other body regions [23]. Finally, validating female athlete experiences within sport by appropriately categorising breast health as a recognised region (Breast) should not be overlooked. There is a high prevalence of breast injuries in contact sports, but they often go unreported [12]. Athletes may be unaware they should report them, but support staff may also be unaware of their occurrence in contact sport [11] and may not know to record them due to a lack of body region specificity and diagnostic codes.

It is worth highlighting that the breast is like other important organs, as it has diagnoses that are clearly injuries, clearly medical conditions and occasionally some diagnoses which are hard to differentiate as an injury or medical condition. For other chest region organs, some resolutions have been as follows: 1) Most lung diagnoses are considered illnesses, but pneumothorax is appropriately considered a chest region injury (and often associated with rib fractures, also a chest region injury) and; 2) Cardiac diagnoses are by contrast all assumed to be medical conditions, including commotio cordis. Commotio is certainly an ambiguous diagnosis in that it is trauma-induced (suggesting that it should be an injury not an illness) but it is also a conduction disorder (inappropriate rhythm) which suggests it should be an illness. The breast has some clearly distinctive diagnoses: a breast haematoma in response to trauma is clearly an injury [17,18], whereas an atraumatic breast lump (either cancerous or benign) is clearly an illness. Infected breast tissue as a sequela of acute or chronic trauma exists in the grey zone of part-injury part-illness.

Where the breast differs from the heart or lungs is that the latter two major organs have a clearly defined “system”. Heart “illness” diagnoses neatly fall into the cardiology system and lung “illness” diagnoses neatly fall into the respiratory system. Breast illness diagnoses are not as clearly or neatly part of the female reproductive system. Medical doctors generally consider the female reproductive system to be the domain of gynaecologists, who do not typically treat breast illnesses. Hence there is an argument to be made for breast exceptionalism, where the same may not be true compared to male reproductive injuries and illnesses. The testes can suffer from injuries (e.g. traumatic rupture) and illnesses (e.g. testicular cancer), but regionally disorders are easier to classify (injuries ​= ​groin region; illnesses ​= ​genitourinary).

A further consideration with respect to a regional code of the letter B (considered for Breast) is that in versions prior to OSIICS 13.0 the B region was designated for Buttock. One of the consensus decisions in 2019 was to merge Lumbar and Buttock regions into a single region. Hence B codes were retired to become L region codes. Whilst, introducing a new anatomical region for Breast (equals region B) may create a degree of ambiguity between OSICS 10 users (B = Buttock) and future version users (B = Breast), this could easily be resolved as body regions rather than letters are typically reported in research. Further, users are encouraged to incorporate up-to-date OSIICS versions in their injury and illness surveillance systems, particularly when conducing health surveillance on female athletes as OSICS 10 would lack the female-specific diagnoses.

Finally, there may be perceived challenges for male athlete coding if Breast was considered a new independent body region. Males do have (somewhat redundant) breast tissue, and they can suffer from breast cancer, albeit this would be exceedingly rare in male athletes. There is also gynaecomastia, a medical condition of male athletes but categorised as an illness rather than injury. Given the relatively small number of distinct diagnoses for male athletes and several diagnoses that are not sex-specific, it would seem feasible to add Breast as a region in systems being used by male athletes. It is also acknowledged that breast injury diagnoses would be relevant for female-to-male and male-to-female transgender athletes, further highlighting the need to incorporate Breast as a region in all systems (men's and women's sports). Such an addition would create minimal burden on users in terms of navigating codes as the Breast region is only one additional category for the top tier of navigation.

The argument for considering the Breast as a distinct body region itself (and not just a body part within the Chest region) is a worthy one and validly introduced by this update. However, we considered that it would and should only be implemented by a future consensus panel that included SMDCS, and only if both systems agreed to simultaneously introduce Breast as a new body region. Therefore, we chose in version 16 to include additional breast diagnoses as part of the Chest region, but with codes that enable an easier shift should a future consensus panel agree that the breast warrants a new region to be created. We also recommend that when reporting injuries using the first letter of the OSIICS (e.g., breakdown of injury rates per body region) that “C” and “CXB” data are presented, particularly for female athletes. This recommendation allows prospective breast injury rates to be determined.

The move to include additional perinatal health diagnoses is a starting point to enable better prospective monitoring of female athletes entering motherhood during their sporting careers [24]. The pregnancy and postpartum periods have a number of associated health problems that may affect an athlete's ability to participate in sport and perform, as well as childbirth-related health problems [9,10,[25], [26], [27], [28]]. Being able to reliably report health problems within these female athlete health domains will allow perinatal policies and guidelines (e.g., maternity leave policy, return-to-sport postpartum guidelines) to be informed by an understanding of the impact the domain has on an athlete's health and participation [20]. Mental health diagnoses codes were included in version 15, but the author group felt it was necessary to include antenatal and postnatal specific anxiety and depression diagnoses in this update, as they are commonly reported in the general perinatal population [29,30] and are distinguishable by the life phase a female athlete is going through.

Some additional ad hoc codes were added for common diagnoses that did not have a specific code. One of these (GCA – Femoro-acetabular impingement) uncovered a diagnosis that had previously been part of OSICS but was inadvertently deleted in recent versions.

A final consideration of this paper is a political one. This update and study for this version was conceived and developed in mid-2024 when there did not appear to be any controversy surrounding publishing research which considered gender-specific issues in sport. While none of the authors of this study are based in the United States of America (USA), we are aware that the 47th President of the USA, in January 2025 took major steps to censor US-government research in sport that has a focus on gender-specific issues [31]. The authorship group of this paper is committed to continuing to report research on sex and gender differences. At a minimum, female-specific analyses and data relating to women in sport have been neglected in the past and deserve ongoing attention and should not be sacrificed in any political attempt to censor research on either DEI (diversity, equity and inclusion) or transgender athlete topics. The controversial issues relating to transgender females and female-assigned intersex athletes participating in women's sport should also continue to give rise to scientific study, as the valid “inclusion” vs “fairness” debate should be resolved with reference to science and data. While recognising that these are easier principles to uphold from outside the USA, we hope all researchers are able to continue publishing on these important topics.

By including the translation of OSIICS into Spanish (and updating the Italian version) there will be greater global reach in the adoption of the OSIICS. Prior to OSIICS version 14, an English language speaker (ideally with medical knowledge) was required to be involved in recording the injuries and illnesses. Removing barriers, such as the English language requirements, to using a classification system can help improve the quality of injury and illness research from countries where English is not the first language. Additionally, it can support clubs to easily undertake internal audits of their injury and illness data by enabling comparisons with consistent diagnostic codes between language versions and published data. Subsequent to this paper, we will work with those who wish to translate OSIICS into additional languages, including French.

## 5 Conclusion

The minor update presented allows more comprehensive injury and illness surveillance of female athletes to be undertaken, aligning with the recent breast injury classification and facilitating female athlete health domains to be explored in large-scale, prospective studies. Whilst the Breast region was not added as a separate body region, it warrants discussion in future consensus updates. Users are encouraged to report the Chest body region as two categories, ‘C’ and ‘CXB’, to support documenting breast injury and illness rates moving forwards. The update also allows great language accessibility.

## Authors’ contributions

JWO is the primary author of the OSIICS; ISM and MMR were involved in updating the female athlete codes; FG and DM were involved in the language translation of the codes; MM, KMC, ER, JO and BC provided oversight of the methods and process of adding code updates. All authors have read and approved the final version of the manuscript and agree with the order of presentation of the authors.

## Declaration of competing interest

The authors declare the following financial interests/personal relationships which may be considered as potential competing interests:Jessica J. Orchard is Editor-in-Chief of JSAMS Plus and is funded by a National Health and Medical Research Council Investigator Grant (No. 2016730).

## References

[1] Bahr R., Clarsen B., Derman W., Dvorak J., Emery C., Finch C. (2020). International olympic committee consensus statement: methods for recording and reporting of epidemiological data on injury and illness in sports 2020 (including STROBE extension for sport injury and illness Surveillance—STROBE-SIIS). Br J Sports Med.

[2] Orchard J.W., Meeuwisse W., Derman W., Hägglund M., Soligard T., Schwellnus M. (2020). Sport medicine diagnostic coding system (SMDCS) and the orchard sports injury and illness classification system (OSIICS): revised 2020 consensus versions. Br J Sports Med.

[3] Orchard J.W., Rio E., Crossley K.M., Orchard J.J., Mountjoy M. (2024). Orchard sports injury and illness classification system (OSIICS) version 15. J Sport Health Sci.

[4] Orchard J., Genovesi F. (2022). Orchard sports injury and illness classification system (OSIICS) version 14 and Italian translation. Br J Sports Med.

[5] Orchard J. (1993). Orchard sports injury classification system (OSICS). Sport Health.

[6] Rae K., Orchard J. (2007). The orchard sports injury classification system (OSICS) version 10. Clin J Sport Med.

[7] Starling L.T., Gabb N., Williams S., Kemp S., Stokes K.A. (2023). Longitudinal study of six seasons of match injuries in elite female rugby union. Br J Sports Med.

[8] Barlow A., Blodgett J.M., Williams S., Pedlar C.R., Bruinvels G. (2024). Injury incidence, severity, and type across the menstrual cycle in female footballers: a prospective three season cohort study. Med Sci Sports Exerc.

[9] Moore I.S., Crossley K.M., Bo K., Mountjoy M., Ackerman K.E., Antero J. (2023). Female athlete health domains: a supplement to the international olympic committee consensus statement on methods for recording and reporting epidemiological data on injury and illness in sport. Br J Sports Med.

[10] Moore I.S., James M.L., Brockwell E., Perkins J., Jones A., Donnelly G.M. (2021). Multidisciplinary, biopsychosocial factors contributing to returnto-running and running-related stress urinary incontinence in postpartum females. Br J Sports Med.

[11] Brisbine B.R., Steele J.R., Phillips E., McGhee D.E. (2020). Breast injuries reported by female contact football players based on football code, player position and competition level. Med Football.

[12] Brisbine B.R., Steele J.R., Phillips E.J., McGhee D.E. (2019). The occurrence, causes and perceived performance effects of breast injuries in elite female athletes. J Sports Sci Med.

[13] Brisbine B.R., Steele J.R., Phillips E.J., McGhee D.E. (2020). Breast pain affects the performance of elite female athletes. J Sports Sci.

[14] Mc Carthy-Ryan M., Perkins J., Donnelly G.M., Caithriona Y., Liston M., Leahy K., Bø K., O'Halloran P., Moore I.S. (2024 Feb 6). Stress urinary incontinence prevalence and risk factors in female rugby players: a common health problem across four nations. BMJ Open Sport Exerc Med.

[15] Dakic J.G., Cook J., Hay-Smith J., Lin K.Y., Frawley H. (2021). Pelvic floor disorders stop women exercising: a survey of 4556 symptomatic women. J Sci Med Sport/Sports Medicine Australia.

[16] Zarei J., Golpira R., Hashemi N., Azadmanjir Z., Meidani Z., Vahedi A. (2025). Comparison of the accuracy of inpatient morbidity coding with ICD-11 and ICD-10. Health Inf Manag.

[17] McGhee D.E., Steele J.R. (2023). Changes to breast structure and function across a woman's lifespan: implications for managing and modeling female breast injuries. Clin Biomech.

[18] Song C.T., Teo I., Song C. (2015). Systematic review of seat-belt trauma to the female breast: a new diagnosis and management classification. J Plast Reconstr Aesthetic Surg.

[19] Brown N., White J., Brasher A., Scurr J. (2014). The experience of breast pain (Mastalgia) in female runners of the 2012 London Marathon and its effect on exercise behaviour. Br J Sports Med.

[20] Donnelly G.M., Moore I.S. (2023). Sports medicine and the pelvic floor. Curr Sports Med Rep.

[21] Wakefield-Scurr J., St John E., Bibby K., Renwick N., Smith N., Hobbs S. (2024). Insights into breast health issues in women's rugby. Eur J Sport Sci.

[22] Burbage J., Cameron L. (2017). An investigation into the prevalence and impact of breast pain, bra issues and breast size on female horse riders. J Sports Sci.

[23] Wakefield-Scurr J., St John E., Bibby K., Renwick N., Smith N., Hobbs S. (2024). Insights into breast health issues in women’s rugby. EJSS (Champaign).

[24] Moore I.S., McCarthy-Ryan M., Palmer D., Perkins J., Verhagen E. (2024). Is your system fit for purpose? Female athlete health considerations for rugby injury and illness surveillance systems. EJSS (Champaign).

[25] Donnelly G.M., Coltman C.E., Dane K., Elliott-Sale K.J., Hayman M., McCarthy-Ryan M.F. (2024). Prioritise safety, optimise success! return to rugby postpartum. EJSS (Champaign).

[26] Kimber M.L., Meyer S., McHugh T.L., Thornton J., Khurana R., Sivak A. (2021). Health outcomes after pregnancy in elite athletes: a systematic review and meta-analysis. Med Sci Sports Exerc.

[27] McNestry C., Killeen S.L., Crowley R.K., McAuliffe F.M. (2023). Pregnancy complications and later life women's health. Acta Obstet Gynecol Scand.

[28] Ceprnja D., Chipchase L., Fahey P., Liamputtong P., Gupta A. (2021). Prevalence and factors associated with pelvic girdle pain during pregnancy in Australian women: a cross-sectional study. Spine.

[29] Horowitz J.A., Goodman J. (2004). A longitudinal study of maternal postpartum depression symptoms. Res Theor Nurs Pract.

[30] Hannon S., Gartland D., Higgins A., Brown S.J., Carroll M., Begley C. (2022). Maternal mental health in the first year postpartum in a large Irish population cohort: the MAMMI study. Arch Womens Ment Health.

[31] JSAMS Plus Editorial Board (2025). Time to draw a line: journal editors must protect scientific independence. JSAMS Plus.

